# Robotic Drop-Coating Graphite–Copper PDMS Soft Pressure Sensor with Fabric-Integrated Electrodes for Wearable Devices

**DOI:** 10.3390/mi16111247

**Published:** 2025-10-31

**Authors:** Zeping Yu, Yunhao Zhang, Lingpu Ge, Daisuke Miyata, Zhongnan Pu, Chenghong Lu, Lei Jing

**Affiliations:** 1 Graduate School of Computer Science and Engineering, University of Aizu, Aizuwakamatsu 965-8580, Japan; d8251103@u-aizu.ac.jp (Z.Y.); d8272109@u-aizu.ac.jp (Y.Z.); c25znpu@u-aizu.ac.jp (Z.P.); luch@sibet.ac.cn (C.L.); 2 Faculty of Information Science and Electrical Engineering, Kyushu University, Fukuoka 819-0395, Japan; gu.lingpu.755@m.kyushu-u.ac.jp; 3 Department of Computer Science and Engineering, University of Aizu, Aizuwakamatsu 965-8580, Japan; daisukem@u-aizu.ac.jp

**Keywords:** flexible pressure sensor, PDMS composite, graphite–copper nanoparticles, robotic drop-coating, textile-integrated electrodes, wearable devices, human–machine interaction

## Abstract

Flexible pressure sensors are essential for wearable electronics, human–machine interfaces, and soft robotics. However, conventional Polydimethylsiloxane (PDMS)-based sensors often suffer from limited conductivity, poor filler dispersion, and low structural integration with textile substrates. In this work, we present a robotic drop-coating approach for fabricating graphite–copper nanoparticle (G-CuNP)/PDMS composite pressure sensors with textile-integrated electrodes. By precisely controlling droplet deposition, a three-layer sandwiched structure was realized that ensures uniformity and scalability while avoiding the drawbacks of conventional full-line coating. The effects of filler loading and graphite nanoparticle (GNP) and copper nanoparticle (CuNP) ratios were systematically investigated, and the optimized sensor was obtained at 40 wt% total fillers with a graphite content of 55 wt%. The fabricated device exhibited high sensitivity in the low-pressure region, stable performance in the medium- and high-pressure ranges, and an exponential saturation fitting with *R*^2^ = 0.998. The average hysteresis was 7.42%, with excellent cyclic stability over 1000 loading cycles. Furthermore, a hand-shaped sensor matrix composed of five distributed sensing units successfully distinguished grasping behaviors of lightweight and heavyweight objects, demonstrating multipoint force mapping capability. This study highlights the advantages of robotic drop-coating for scalable fabrication and provides a promising pathway toward low-cost, reliable, and wearable soft pressure sensors.

## 1. Introduction

With the rapid development of wearable devices in fields such as care monitoring, human-machine interaction, and motion detection, the demand for flexible pressure sensors has been steadily increasing [1,2,3]. These sensors are required not only to exhibit high sensitivity and fast response characteristics but also to possess excellent flexibility, stability, and low-cost manufacturability to meet the needs of diverse and complex application scenarios [4,5]. In particular, in areas such as smart wearables and soft robotics, flexible pressure sensors capable of detecting changes in external pressure in real time and with high accuracy have become a key enabler technology [6,7].

Polydimethylsiloxane (PDMS), because of its flexibility and biocompatibility, has become one of the most commonly used matrix materials for flexible sensors [8]. However, PDMS itself is electrically insulating and thus requires the incorporation of conductive fillers to achieve electrical responsiveness [9]. Carbon-based materials (such as Graphite Nanoparticles (GNPs), Graphene Nanoparticles, and Carbon Nanotubes) and metallic particles (such as Copper Nanoparticles (CuNPs) and Silver Nanoparticles) have attracted widespread attention in recent years as common conductive fillers for flexible pressure sensors. Carbon-based materials generally exhibit excellent flexibility, chemical stability, and good process compatibility; however, their intrinsic electrical conductivity is relatively limited [10,11]. In contrast, metallic particles possess superior electrical conductivity but often suffer from issues such as oxidation, agglomeration, and poor dispersion within polymer matrices [12,13]. To overcome the limitations of single-component fillers, carbon–metal composite conductive fillers have emerged as a research hotspot. Through synergistic effects, these compounds can achieve balanced improvements in conductivity, mechanical compliance, and long-term stability [14,15]. Among various metallic fillers, copper is an attractive filler owing to its high conductivity and low cost compared to noble metals. Although prone to oxidation and agglomeration, copper nanoparticles can enhance electrical pathways in polymer composites [16]. When combined with carbon-based particles, they offer a balance between conductivity, stability, and flexibility.

At the same time, the structural design of the sensors is also a critical factor that determines whether they can achieve long-term and stable operation in wearable applications [17,18]. A common approach is to first fabricate the sensing unit using flexible substrate materials and then connect it to the signal processing circuit via external wires [19]. Although this configuration can meet the basic requirements of signal acquisition, it still suffers from significant drawbacks in practical applications. First, the additional wiring inevitably increases the overall size and weight of the device, thereby reducing its flexibility and comfort in wearable scenarios. Second, the wires are prone to mechanical fatigue or even fracture under dynamic bending, stretching, and prolonged use, which severely compromises the stability and service life of the device [17,20,21]. Therefore, how to simplify the device structure and enhance the overall integration while maintaining sensing performance has become a key issue in the design of flexible wearable sensors [18].

To address the strong dependence of conventional flexible sensors on external wiring, various novel structural design strategies have been proposed in recent years. One approach is based on flexible electronic printing or spraying structures, in which conductive inks or functional nanomaterials are directly patterned and deposited onto flexible substrates to simultaneously form both the electrodes and the sensing unit. This method offers high design freedom and scalability, enabling large-area and low-cost fabrication; however, challenges remain in terms of long-term stability and durability under dynamic stretching conditions [22,23]. Another approach is to directly coat sensing materials onto conductive textile filaments, thereby achieving integration of the sensing layer with the electrodes. This strategy can reduce reliance on external wiring and improve the integration and flexibility of the sensor, but the coating process often results in a significant increase in the surface friction coefficient of the conductive filaments. Consequently, issues such as blockage and abrasion during routing, weaving, and large-scale processing arise, which limit its applicability in mass production [16,24]. It should be noted that although both of these methods show promising potential in terms of material design and integration, their implementation generally requires expensive and large-scale production equipment, which to some extent constrains their feasibility for practical industrialization and widespread application [25].

To address these limitations, this study proposes a novel three-layer sandwiched flexible pressure sensor structure. Specifically, a robotic drop-coating process is employed to precisely deposit individual droplets of a graphite–copper nanoparticle (G-CuNP)/PDMS composite conductive material onto the lower textile electrode, followed by covering with an upper textile electrode to form a stable sandwich configuration. Compared with conventional full-line coating methods, this strategy effectively avoids the increased surface friction that hinders weaving and large-scale processing, while maintaining good overall flexibility and scalability of the device. On this basis, the performance of the proposed structure is further investigated under different graphite nanoparticle (GNP) and copper nanoparticle (CuNP) ratios, in order to validate the effectiveness of the new architecture and to explore feasible pathways for optimizing sensing sensitivity and stability.

The main contributions of this work can be summarized as follows:

- Proposing a novel three-layer sandwiched flexible pressure sensor architecture that combines textile electrodes with a conductive PDMS-based composite layer.
- Developing a robotic drop-coating strategy that enables precise and scalable fabrication of composite sensing layers with uniform morphology.
- Systematically investigating different ratios of graphite nanoparticles (GNPs) and copper nanoparticles (CuNPs), and identifying an optimal filler loading that balances conductivity, sensitivity, and processability.
- Demonstrating the feasibility of distributed pressure sensing by integrating multiple sensors into a hand-shaped matrix, which can reliably distinguish grasping tasks with objects of different weights.

## 2. Materials and Methods

The G-CuNP/PDMS composite conductive material was prepared as follows: PDMS (Sylgard 184, Dow Corning Corporation, Midland, MI, USA) was used as the matrix and thoroughly mixed with its curing agent at a mass ratio of 10:1 [26]. GNPs (400–1200 nm, US Research Nanomaterials, Houston, TX, USA) and CuNPs (580 nm, US Research Nanomaterials) were subsequently incorporated to construct the carbon–metal composite conductive system. During preparation, the viscosity of the mixture was continuously monitored using a viscometer (VISCOQC100-R, Anton Paar, Graz, Austria) to ensure suitable rheological properties for the robotic drop-coating process. To achieve optimal dispensing behavior, the viscosity was precisely adjusted to approximately 4000 mPa·s by adding an appropriate amount of siloxane (KF-96L-0.65CS, Shin-Etsu Chemical Co., Ltd., Tokyo, Japan) [27]. The mixture was then centrifuged at 3000 rpm for 2 min to promote homogeneous dispersion of the conductive fillers. To investigate the influence of filler loading and composition on sensor performance, the total filler content was adjusted within the range of 30–50 wt%, and the GNP content within the conductive fillers was systematically varied to 45, 55, and 65 wt% [16]. Finally, the composite solution was degassed under vacuum (30 Pa) to remove air bubbles and stored for subsequent sensor fabrication.

**Figure 1:**
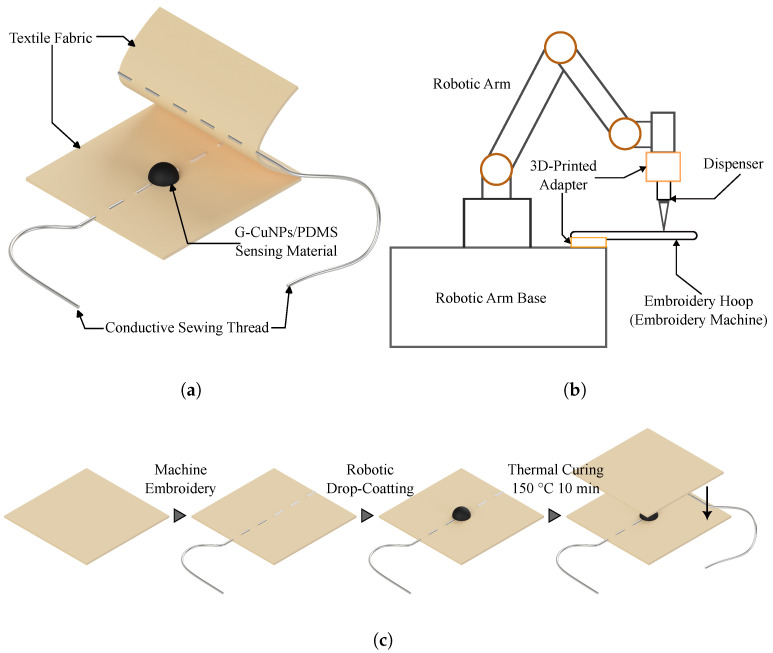
(**a**) Structure of the sensor. (**b**) Schematic illustration of the robotic drop-coating process. (**c**) Fabrication sequence of the sensor.

As shown in Figure 1a, the sensor proposed in this study adopts a three-layer sandwiched structure composed of an upper and a lower textile electrode and an intermediate composite sensing layer. Figure 1c illustrates the fabrication process of the sensor. The textile electrodes were fabricated first: a 100% polyester fabric (heat resistant up to 200 °C) was used as the substrate, and conductive threads (AGposs^®^, Mitsufuji Corporation, Kyoto, Japan) were stitched onto its surface via an embroidery process to form the electrode patterns. Specifically, the required electrode geometries were designed using companion software (ESY1011, Brother Industries, Ltd., Nagoya, Japan) and automatically stitched by an embroidery machine (PR1055X, Brother Industries), yielding textile electrodes that are both flexible and highly conductive. Compared with directly using commercial conductive fabrics, this method allows for flexible control over electrode geometry and distribution, making it more suitable for customized sensor development.

The composite sensing layer was then deposited using a robotic drop-coating process. As shown in Figure 1b, the setup consisted of a six-degree-of-freedom robotic arm (FR5, FAIR Innovation (Suzhou) Robot Systems Co., Ltd., Suzhou, China), a high-precision dispenser (MSIC16-02, ICOMES Lab Co., Ltd., Morioka, Japan), and two custom 3D-printed adapters. The upper orange adapter provided rigid mounting and coaxial alignment between the robot and the dispenser to ensure stable and precise droplet deposition; the lower orange adapter was used to mount the Embroidery Hoop. During the embroidery step, the fabric must be fixed by an Embroidery Hoop. To avoid errors introduced by re-clamping after embroidery, the same Embroidery Hoop was directly interfaced with the dispensing fixture so that the fabric could proceed to the drop-coating step without removal. This ensured consistent fabric tension and planarity throughout, effectively improving the accuracy and repeatability of the dispensing process.

During dispensing, the dispenser parameters were set as follows: a droplet volume of 7μL was ejected per shot, followed by aspiration of 3μL to prevent material buildup at the nozzle tip; a 20 G nozzle (inner diameter 0.61 mm) was employed; the nozzle-to-fabric standoff distance was maintained at approximately 1 mm to stabilize droplet morphology and achieve uniform deposition. The deposited layer was subsequently cured at 150 °C for 10 min to form a stable composite sensing layer. For applications involving natural-fiber textiles with relatively low thermal resistance, a lower-temperature curing condition (e.g., 100 °C for 30 min) can also be employed to ensure substrate compatibility and prevent thermal degradation. Finally, another pre-fabricated textile electrode was placed on top and secured by sewing to obtain the complete sandwiched flexible pressure sensor.

The surface morphology and internal structure of the devices were examined using an optical microscope (JL246PS, JOYALENS, Xinmi, China) and a scanning electron microscope (SEM)(JSM-5900LV, JEOL Ltd., Akishima, Japan). The electrical performance of individual sensors was evaluated by measuring the resistance variation under applied pressure using an impedance analyzer (ZM2372, IET Labs, Roslyn Heights, NY, USA) under small-signal AC excitation. Pressure loading was applied by a mechanical testing platform (ZTA-500, IMADA Co., Ltd., Toyohashi, Japan)(load capacity: 500 N) with a controllable loading rate ranging from 0.5 to 300 mm/min. Data acquisition and control were implemented via LabVIEW software to ensure the synchronization between mechanical loading and electrical measurements. For the assembled hand-shaped sensor matrix, a custom-designed circuit was employed for signal acquisition and processing, enabling real-time detection and recording of multipoint pressure.

## 3. Results and Discussion

**Figure 2:**
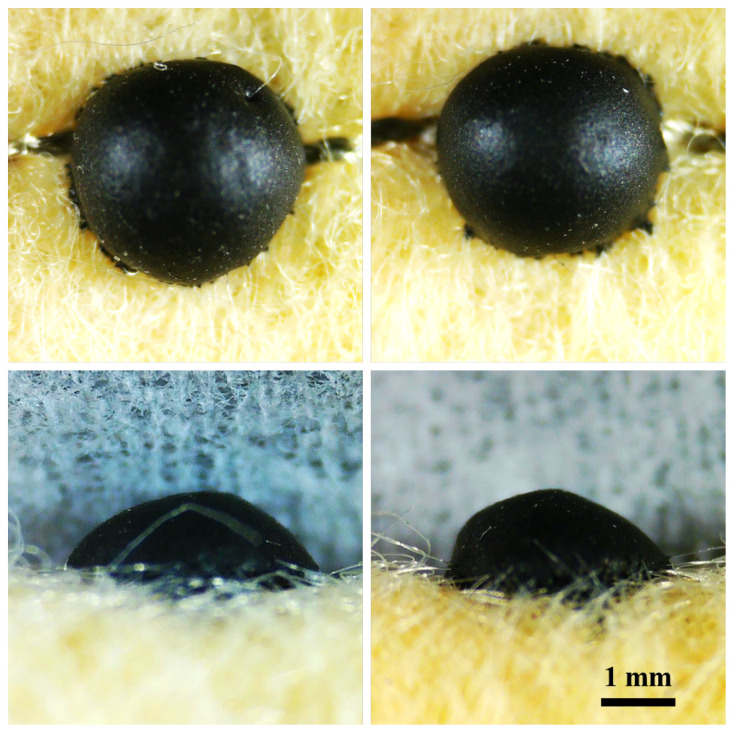
Optical microscopy images of the fabricated pressure sensors.

As shown in Figure 2, the diameter of the sensors was measured under an optical microscope across multiple samples. The statistical analysis yielded a value of 3.12 ± 0.13 mm (*n* = 27), with a range of 3.00–3.36 mm and a coefficient of variation of 4.1%. This result indicates that the proposed robotic drop-coating process can effectively control droplet size, thereby ensuring high consistency and reproducibility of the sensor diameter during batch fabrication. Similarly, the thickness of the sensing layer was determined to be 1.42 ± 0.06 mm (*n* = 27), with a range of 1.34–1.50 mm and a coefficient of variation of 4.5%. The relatively small variation in thickness further demonstrates the stability and controllability of the automated drop-coating method in regulating film thickness. Overall, these results confirm that the developed fabrication process provides uniform geometrical dimensions, laying a reliable foundation for subsequent performance characterization and large-scale applications.

**Figure 3:**
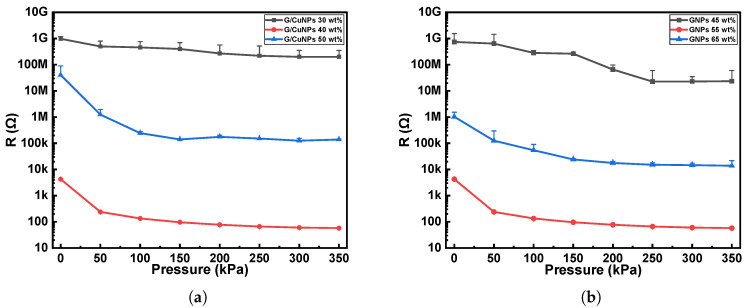
Sensor responses under different weight ratios: (**a**) Response characteristics with GNPs content fixed at 55 wt% and varying weight ratios of conductive materials. (**b**) Response characteristics with conductive material content fixed at 40 wt% and varying GNPs weight ratios.

**Figure 4:**
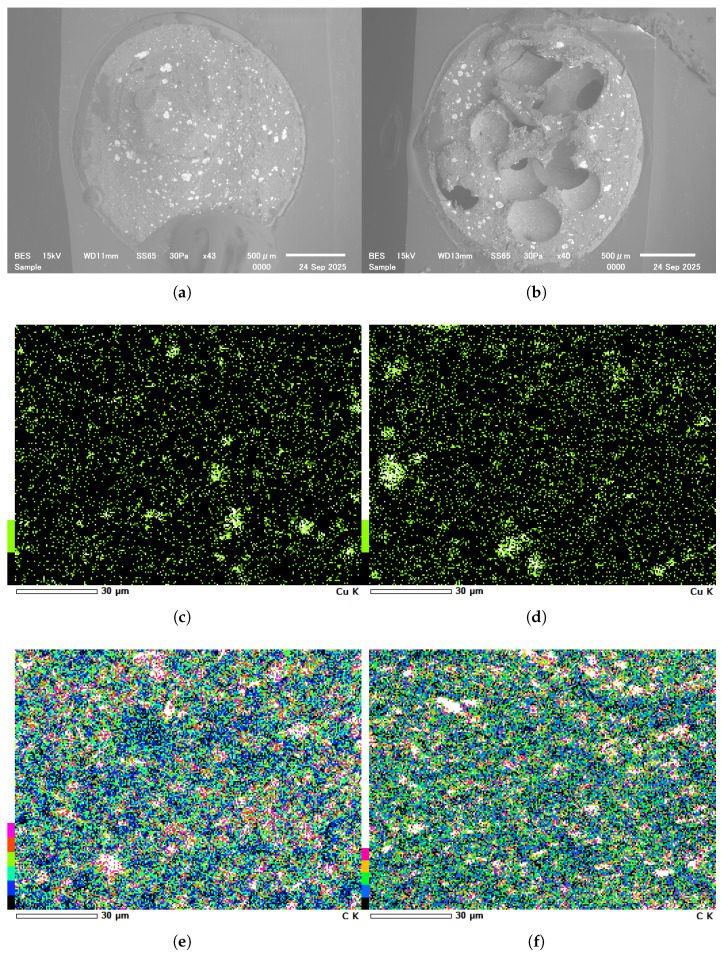
SEM images of the composite sensing layer under different material ratios: (**a**) Morphology with siloxane diluent content of 5 wt%. (**b**) Morphology with siloxane diluent content of 20 wt%. (**c**) Distribution of CuNPs at GNPs content of 55 wt%. (**d**) Distribution of CuNPs at GNPs content of 45 wt%. (**e**) Distribution of GNPs at 55 wt%. (**f**) Distribution of GNPs at 45 wt%.

As shown in Figure 3, the performance of the proposed sensor structure was systematically evaluated under different weight ratios. Figure 3a presents the responses when the conductive fillers accounted for 30 wt%, 40 wt%, and 50 wt% of the total composite, while the GNPs content within the conductive fillers was fixed at 55 wt%. The results indicate that at 30 wt%, the conductive fillers failed to form an effective conductive network within the PDMS matrix, leading to an extremely high device resistance with almost no response to the applied pressure. When the weight ratio increased to 40 wt%, the conductive network gradually became continuous, and the sensor exhibited significantly enhanced sensitivity and improved linearity, suggesting that the fillers were more uniformly distributed and capable of forming stable resistance pathways under pressure. However, when the filler content was further increased to 50 wt%, the overall resistance did not decrease as expected but instead increased, accompanied by pronounced signal fluctuations and instability in the low-pressure region. SEM morphology images (Figure 4a,b) revealed that, compared with the 40 wt% sample, the 50 wt% sample contained a larger number of void-like cavities. This phenomenon may be attributed to two factors. On one hand, the excessive filler ratio dramatically increased the viscosity of the composite solution, thereby impairing its flowability and uniformity on the textile electrodes. To maintain processability, a higher amount of siloxane diluent had to be introduced. However, excessive siloxane tended to volatilize rapidly during curing, resulting in localized voids that disrupted the continuity of the conductive network. On the other hand, the introduced cavities may act as micro-buffer chambers during pressure loading, preventing effective stress transfer to the conductive pathways and thereby reducing sensitivity in the low-pressure range and introducing noise.

Furthermore, the performance of samples with GNPs contents of 45 wt%, 55 wt%, and 65 wt% within the conductive fillers was also evaluated, as shown in Figure 3b. The results demonstrated that when the GNPs ratio was 45 wt%, the device exhibited negligible conductivity and responded poorly to pressure. SEM mapping analysis (Figure 4c,d) together with the quantitative results in Table 1 showed that, although the addition of CuNPs slightly reduced the fraction of filler-deficient regions by only about 1.9%, this limited improvement was accompanied by a pronounced increase in the agglomerated domains. This indicates that the enhanced Cu content primarily promoted particle clustering rather than forming effective interconnections between GNPs, thereby hindering the establishment of a continuous conductive network. In addition, the mapping results (Figure 4e,f) and the corresponding data in Table 1 revealed that the reduction of GNP content markedly increased the proportion of filler-deficient regions from about 12.1% to 16.8%, while the average area of these regions also expanded from 9.62 px^2^ to 11.35 px^2^. Such enlargement of non-conductive domains disrupted the uniformity of the conductive pathways and further degraded the overall sensing performance. When the GNP content was further increased to 65 wt%, the composite exhibited excessive viscosity and poor processability, resulting in unstable conductive networks and no further improvement in sensing performance.

In summary, this study finally adopted 40 wt% of total filler loading and 55 wt% GNPs content within the conductive fillers to fabricate the pressure sensors, in order to achieve a balance between superior electrical performance and favorable processability.

In this study, the relative resistance change of the sensor was expressed as $-\Delta R/R_{0}$, where $R_{0}$ is the initial resistance. Since the resistance increased with applied pressure during the loading process, directly using $\Delta R/R_{0}$ would result in negative values, which is unfavorable for curve comparison and reader comprehension. Therefore, $-\Delta R/R_{0}$ was uniformly adopted in this work to represent the relative resistance change, so that the response curves exhibit an overall positive growth trend, facilitating subsequent fitting and comparative analysis.

**Figure 5:**
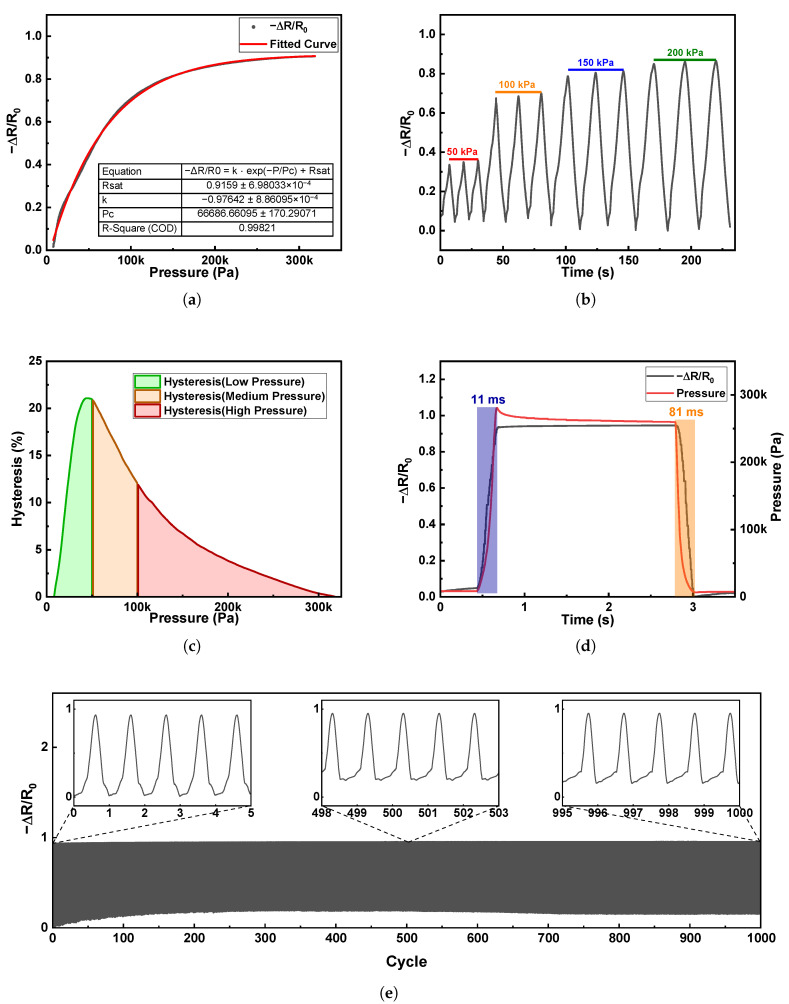
Performance characterization of the pressure sensor: (**a**) Fitted response curve of the sensor under applied pressure. (**b**) Repeatability of the sensor response under different pressures. (**c**) Hysteresis ratio measured at various pressure levels. (**d**) Response and recovery time during loading and unloading processes. (**e**) Durability test of the sensor over 1000 loading–unloading cycles.

As shown in Figure 5a, the experimental results reveal that the relationship between $-\Delta R/R_{0}$ and the applied pressure follows an exponential saturation behavior: a rapid resistance change occurs in the low-pressure region, while the response gradually stabilizes in the medium- to high-pressure range. Each data point represents the grand average obtained from 9 independently fabricated sensors, with 5 repeated loading–unloading cycles measured for each sensor, to ensure statistical reliability. The relationship between the relative resistance change and the applied pressure was analyzed and fitted using the exponential saturation model, which can be mathematically expressed as

(1) $$-\frac{\Delta R}{R_{0}}=k\cdot e^{-P/P_{c}}+R_{\mathrm{sat}}$$

where *P* is the applied pressure, $R_{\mathrm{sat}}$ is the saturation value, *k* is the amplitude factor, and $P_{c}$ is the characteristic pressure constant. The fitting result obtained from Origin yielded a coefficient of determination $R^{2}=0.998$. The fitted curve exhibited excellent agreement with the experimental data, confirming that the exponential saturation model can accurately describe the response characteristics of the sensor over the entire pressure range. According to the commonly adopted testing standards for wearable pressure sensors, the loading range was divided into three regions for analysis: low pressure (0–50 kPa), medium pressure (50–100 kPa), and high pressure (>100 kPa) [28]. From the perspective of response mechanisms, in the low-pressure region, the resistance changes rapidly with increasing pressure due to the significant enhancement of tunneling effects between conductive fillers during the initial compression stage. In contrast, in the medium- and high-pressure regions, as the conductive pathways gradually reach saturation and the compressibility of the PDMS matrix becomes restricted, the resistance change slows down and approaches the saturation value. This trend is consistent with previous reports on PDMS-based flexible pressure sensors, further validating that the fabricated device exhibits high sensitivity in the low-pressure range and excellent mechanical stability in the medium- to high-pressure regions.

Figure 5b shows the dynamic resistance response of the sensor under different applied pressures, where the normalized variation $-\Delta R/R_{0}$ is plotted as a function of time. Distinct and stable resistance changes are observed at 50, 100, 150, and 200 kPa, demonstrating that the sensor exhibits clear pressure-dependent responses. With increasing applied pressure, the response amplitude gradually increases, indicating the enhanced compressive deformation of the composite sensing layer. Moreover, the curves exhibit fast and repeatable transitions between loading and unloading states, suggesting good reversibility and stability of the sensor during cyclic operation.

The hysteresis performance of the sensor is illustrated in Figure 5c. The test results show that the overall average hysteresis ratio of the sensor was 7.42%, with average values of 13.48%, 16.34%, and 4.19% in the low-, medium-, and high-pressure ranges, respectively. The maximum hysteresis ratio appeared at approximately 45 kPa, reaching 21.07%. Compared with single carbon-based composite/PDMS pressure sensors reported in the literature (with hysteresis ratios of about 8–13%) [29,30,31], the sensor fabricated in this work exhibited a much lower hysteresis in the high-pressure range, indicating excellent mechanical stability. However, the hysteresis ratios in the low- and medium-pressure ranges were relatively high, which can be mainly attributed to the insufficient resilience of the textile substrate in the composite structure. This limited rebound behavior leads to larger energy dissipation during loading and unloading cycles. Therefore, future work may focus on optimizing the selection of textiles with improved elastic recovery as the electrode substrate, in order to further reduce the hysteresis in the low- and medium-pressure ranges and enhance the overall sensing performance.

As shown in Figure 5d, the response times of the sensor during loading and unloading were investigated. To minimize the influence of the mechanical testing platform itself, the loading and unloading speed was set to 150 mm/s, so that the input pressure profile approximated a step signal. The loading response time is defined as

(2) $$t_{response,\mathrm{load}}=t_{R,90\%}-t_{P,90\%}$$

where denotes $t_{P,90\%}$ the time at which the input pressure signal reaches 90% of its steady-state value, and $t_{R,90\%}$ denotes the time at which the sensor output signal reaches 90% of its steady-state value. This time difference represents the dynamic delay required for the sensor to reach the same level as the input during the loading process. Similarly, the unloading response time is defined as

(3) $$t_{response,\mathrm{unload}}=t_{R,10\%}-t_{P,10\%}$$

where $t_{P,10\%}$ and $t_{R,10\%}$ correspond to the times when the input pressure and the sensor output decrease to 10% of their steady-state values, respectively. This parameter characterizes the recovery speed of the sensor during the unloading process. By employing this percentage-matching method, the influence of the actuator’s finite rising and falling times can be effectively eliminated, thereby providing a more accurate characterization of the intrinsic dynamic performance of the device. The experimental results demonstrate that the loading response time of the sensor is 11 ms, whereas the unloading response time is 81 ms, indicating that the loading process is significantly faster than the unloading process. This phenomenon can be mainly attributed to the viscoelasticity of the composite sensing layer and interfacial effects: during loading, the external force directly drives conductive fillers to rapidly approach each other and form conductive networks, leading to a rapid decrease in resistance; in contrast, during unloading, the viscoelastic recovery of the polymer matrix is restricted by chain relaxation dynamics, while interfacial adhesion and friction between conductive fillers further delay the breakdown and recovery of conductive pathways, resulting in a markedly slower response.

As shown in Figure 5e, the sensor was subjected to 1000 cyclic loading–unloading tests at 350 kPa to evaluate its durability and stability. The experimental results demonstrate that the sensor maintained a stable response curve throughout the entire test, and even after 1000 cycles, its sensitivity and response profile remained well preserved, indicating excellent cyclic stability. It is noteworthy that during approximately the first 100 cycles, the baseline resistance of the sensor exhibited a slight drift. This phenomenon can be mainly attributed to the presence of microscopic gaps between the textile electrodes and the composite sensing layer in the newly fabricated sensor. During the initial loading cycles, the textile fibers were gradually compacted, leading to a slow shift of the baseline. With increasing cycle numbers, these gaps were progressively compressed and closed, resulting in more stable interfacial contact and, consequently, a stabilized baseline.

**Figure 6:**
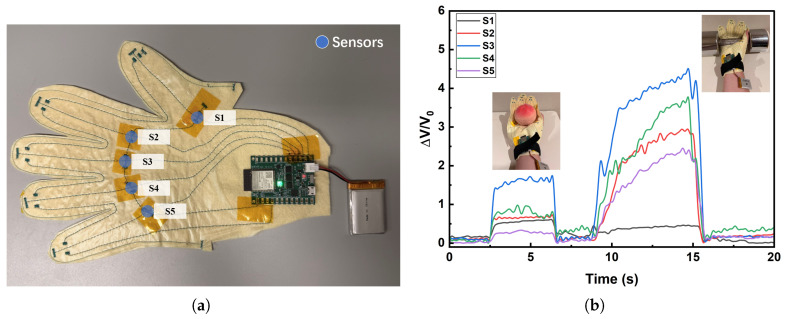
(**a**) Photograph of the hand-shaped pressure sensor glove. The blue circles indicate the positions where pressure sensors are integrated, arranged from top to bottom as S1 to S5. (**b**) Normalized voltage responses ($\Delta V/V_{0}$) of the glove when grasping a lightweight object (peach) and a heavyweight object (dumbbell).

The hand-shaped pressure sensor glove was fabricated by integrating five pressure sensors (blue circles in Figure 6a) onto a textile substrate, with their positions distributed across the palm and the pulp of the thumb. This layout allows representative force distributions to be captured during grasping tasks. The detailed fabrication process and circuit implementation have been described in our previous work, where the specific conversion relationship between resistance and voltage was also provided [32].

Figure 6b shows the normalized voltage responses ($\Delta V/V_{0}$), which are inversely related to the resistance changes of the sensors. When grasping the lightweight peach, only moderate increases are observed. The response of S3 is the most pronounced, reaching about 1.5, while S1, S2 and S4 show smaller but noticeable increases (approximately 0.5–1). In contrast, S5 exhibits almost no response, indicating that the outer palm region near the little finger bears minimal load when handling a lightweight object.

In contrast, when grasping the heavyweight dumbbell, the sensor outputs increase sharply. S3 exhibits the highest response (exceeding 4.5), followed by S4 (approximately 3.8), S2 (approximately 3.0), and S5 (approximately 2.5). S1 shows only a weak signal and remains close to the baseline. These results clearly indicate that the palm-region sensors (S2, S3, S4, and S5) play a critical role during heavy-load grasping. The weak response of S1 can be attributed to the difference in contact mechanics: while the spherical peach engages the thumb pulp and generates a moderate signal, the cylindrical handle of the dumbbell shifts the primary loading points to the central palm regions, resulting in minimal activation of S1.

These contrasting results highlight two important aspects of the glove’s performance. First, the system is highly sensitive to changes in gripping force, as reflected by the large difference in response amplitude between the peach and dumbbell tests. Second, the sensor array provides spatially resolved information on how contact forces are distributed across the hand, which is strongly influenced not only by object weight but also by object geometry. For instance, the nearly absent signal from S5 in the peach test indicates that the outer palm contributes little when grasping a small spherical object, whereas its strong activation in the dumbbell test reflects the broader engagement of this region when holding a cylindrical handle. Similarly, the discrepancy in S1’s response between the two conditions can be attributed to the difference in contact mechanics: while the thumb pulp is partially involved in stabilizing the peach, it is largely bypassed during cylindrical handle grasping, leading to minimal activation.

## 4. Conclusions

In this study, we developed a robotic drop-coating strategy for the fabrication of PDMS-based composite pressure sensors incorporating graphite and copper fillers with textile-integrated electrodes. The automated process enabled precise droplet deposition, resulting in uniform device dimensions and reproducible performance. With optimized filler loading (40 wt% total filler with 55 wt% GNPs), the fabricated sensors exhibited a high sensitivity in the low-pressure region and a wide effective pressure detection range up to 300 kPa (most reported flexible pressure sensors operate below 100 kPa [33]). The sensors showed an average hysteresis of 7.42%, indicating good mechanical stability compared with conventional carbon-based sensors(with hysteresis ratios of about 8–13% in other studies). Moreover, the sensors demonstrated fast response and recovery times of approximately 11 ms and 81 ms (typical flexible pressure sensors show response/recovery times of about 20–100 ms), and stable outputs at higher pressures [29,30,31,34,35]. The sensors also maintained excellent durability over 1000 loading–unloading cycles, confirming their mechanical robustness. Furthermore, the integration of five sensors into a hand-shaped matrix validated the feasibility of distributed pressure mapping, as the system reliably distinguished between light and heavy grasping tasks with high repeatability. These results highlight the effectiveness of the robotic drop-coating method and its potential for scalable manufacturing of wearable sensing systems.

Despite the promising performance, some limitations remain. The textile electrodes used in this study exhibited noticeable hysteresis in the low- and medium-pressure ranges, which may reduce accuracy for subtle force detection. This hysteresis mainly originates from the limited elastic recovery of the textile substrate, which undergoes residual deformation after cyclic loading. Although dynamic mechanical analysis (DMA) was not performed in this work, such quantitative evaluation and the use of more elastic textile materials could help mitigate this effect in future studies. In addition, baseline drifts were occasionally observed during long-term testing, suggesting the need for improved encapsulation and environmental stability. Moreover, the slow baseline shift during the initial loading cycles was mainly caused by the fluffy and loosely structured textile substrate, which tends to settle mechanically under repeated compression before reaching a stable state. In future work, more compact and tightly woven fabrics will be adopted to improve structural stability and minimize this effect. Future work will therefore focus on optimizing textile substrates and electrode materials to minimize hysteresis, enhancing packaging strategies for long-term reliability, and extending the robotic drop-coating approach to other types of soft electronics, such as stretchable strain sensors and multimodal wearable devices.

## Figures and Tables

**Table 1:** Quantitative analysis of filler-deficient and agglomerated regions.

Sample	Filler-Deficient Regions(%) & Average Area (px^2^)	Agglomerated Regions(%) & Average Area (px^2^)	Figure
CuNPs(55 wt% GNPs)	83.056%	7.077%	Figure 4c
	-	31.92	
CuNPs(45 wt% GNPs)	81.154%	9.233%	Figure 4d
	-	42.85	
CNPs(55 wt% GNPs)	12.083%	13.146%	Figure 4e
	9.62	18.09	
GNPs(45 wt% GNPs)	16.805%	13.624%	Figure 4f
	11.35	17.83	

## Data Availability

The data presented in this study are available from the corresponding author upon request.

## Abbreviations

The following abbreviations are used in this manuscript:

PDMS	Polydimethylsiloxane
SEM	Scanning electron microscope
GNPs	Graphite nanoparticles
CuNPs	Copper nanoparticles
G-CuNPs	Graphite–copper nanoparticles
DMA	Dynamic mechanical analysis
